# Face coverings: Considering the implications for face perception and speech communication

**DOI:** 10.1186/s41235-023-00479-w

**Published:** 2023-04-21

**Authors:** Karen Lander, Gabrielle H. Saunders

**Affiliations:** 1 Division of Psychology, Communication and Human Neuroscience, School of Health Sciences, University of Manchester, Manchester, M13 9PL UK; 2 Manchester Centre for Audiology and Deafness, and NIHR Manchester Biomedical Research Centre - Hearing Health theme, University of Manchester, Manchester, UK

Cognitive Research: Principles and Implications has released a comprehensive collection of thirty articles on the impact of face coverings for face perception and speech communication. This thematic series was motivated by the global COVID-19 pandemic in 2020, when many governments around the world required or strongly recommended the wearing of face coverings (masks) in public spaces. It seems likely that the use of face coverings will continue, albeit in a more selective manner, even now that the acute phase of the COVID-19 pandemic appears to be over.

Research on the impacts of face coverings on communication increased massively in 2020, with early work establishing, unsurprisingly, that masks hinder the recognition of identity and expression. Masks also impair speech communication and understanding and as a consequence can negatively affect psychosocial well-being. Papers included in this thematic collection represent the second phase of research, where more nuanced effects are investigated and the processes underpinning the effects found are explored. Papers included in this thematic series can be grouped by topic.

A number of papers explored the role of mask wearing in identity recognition, learning and matching. Previous research established that wearing a mask impairs recognition (see for example, Carragher & Hancock, 2020; Freud et al., 2020), however the extent and range of this effect and its mechanisms are less well understood. To this aim, in this thematic series, the detrimental effects of masks were investigated in children (Stajduhar & Freud, 2022) and in adults with Autistic Spectrum Disorder (Tso et al., 2022). Despite the detrimental effect of masks on famous face identification (Wong & Estudillo, 2022) familiarity-detection could still occur (Carlow et al., 2022). Research showed that it was harder to remember not only the identity of unfamiliar faces but whether or not that unfamiliar face wore a mask (Kollenda & de Haas, 2022*)*.

For face identity matching, Bennetts et al. (2022) found decreased performance with masks and sunglasses but no impact of face mask exposure. Interestingly, although Estudillo and Wong (2022) found that face masks disrupted matching performance, for ‘match’ trials there was better performance when both faces were wearing masks rather than just one. Similarly, in a forensic context, identification of a masked perpetrator was increased when a masked line-up was also used (target present condition; Manley et al., 2022). The importance of congruity between stimuli is highlighted. This is consistent with the paper by Thorley et al. (2022) who found that eyewitnesses may struggle when perpetrators wear face masks during offences (but not at identification). They also found inaccurate age estimates of people who wear face masks (also see Ganel & Goodale, 2022; Wong & Estudillo, 2022). The paper by Carragher et al., (2022) suggests that some of the deficit in masked face matching may be alleviated by feature-based training.

The relationship between stimuli at face learning and recognition was explored by Hsaio et al. (2022) using an eye-tracking methodology. Results showed that eye movements during recognition were mainly driven by the mask condition during recognition but not that during learning and those who adjusted their strategy according to the mask condition difference between learning and recognition had better performance. Further work has explored the dynamic interplay between mask wearing at encoding and recognition (Garcia-Marques et al., 2022).

In a second group, several papers examined the effect of wearing a face mask on the ability to recognise emotion from facial expressions. Previous research has shown that face masks impair the ability to perceive social information and the recognition of emotion (see review by Pavlova & Sokolov, 2022). Whilst research shows that different mask designs had equivalent detrimental effects on recognition accuracy (Blazenkova et al., 2022), some people may be more negatively impacted by masks than others (Swain et al., 2022). Interestingly, some of the negative effects of face masks on the understanding of emotional states were reduced by the use of transparent masks (McCrackin et al., 2022).

Grenville and Dwyer (2022) found that there was an overall emotion recognition accuracy for faces shown without masks compared to when shown wearing them, but this effect varied by emotion (advantage without masks in disgust, happiness, and sadness; no effect for neutral; lower accuracy without masks for anger and fear). Using a similar static-based face emotion recognition task, Rinke et al. (2022) showed that the impairment was largest for disgust, followed by fear, surprise, sadness, and happiness. It was not significant for anger (also see Wong & Estudillo, 2022*)* or neutral expressions. Here, they concluded that participants were likely to confuse emotions that share activation of the visible muscles in the upper half of the face. Using dynamic face stimuli, Henke et al. (2022) also found that face masks reduced emotion recognition accuracy and confidence in both younger and older participants. A few papers looked at other aspects of faces including gender decisions (Wong & Estudillo, 2022) and face attractiveness (Hies & Lewis, 2022*; *Pazhoohi et al., 2022*).*

The final set of papers looked at ways in which face masks affected communication in terms of understanding, interactions and interpretation. In a large survey conducted in Australia, Galvin et al., (2022) found that face masks negatively affected the quality of communication, feelings about communication, and led to increased fatigue and frustration and decreased time spent communicating. Lee et al. (2022) found similar results for communication in healthcare settings, emphasizing the added cognitive load experienced by both patients and providers, and associated decreased clinical efficiency. Crinnion et al., (2022) showed problems with masked speech recognition did not change over the course of a year. Sinagra and Wiener (2022) noted that masks can make it more difficult to understand the intended intonation and emotional meaning of speech, while Gutierriz-Sigut et al. (2022); Lee et al. (2022) and Lau et al. (2022) all noted that the negative impacts of face masks were greater for people who are deaf or hard of hearing. At a societal level, the data of Krishna et al., (2021) collected via an online approach-avoidance task indicated that attitudes towards masks and COVID-19 anxiety influenced attitudes towards mask wearers*.*

A number of studies investigated strategies and interventions to manage the impacts of mask wearing. Poon and Jenstad (2022) identified several practical ways to support people who are D/deaf or hard of hearing. These included using transparent masks, improved guidance on when to wear masks, and educating the public about ways to communicate clearly when wearing a mask. Finally, Gutz et al., (2022) showed that while speaking loudly or clearly can compensate for the presence of a mask, such strategies require an increased physical and cognitive effort by the speaker.

This thematic series has raised many new questions about how face perception and communication is influenced by face coverings. For example, we must extend research to include the realistic scenario where masks are worn on dynamic faces. Here the way the face moves behind the mask may give clues about both the identity of the person shown and the expression they are displaying. Individual characteristic movements are known to aid familiar face recognition (Lander et al., 1999) and such parameters may also be present from a face wearing a mask. Similarly, other individual differences in the wearer and mask (for example, size of face, relative size and location of face mask, way face mask is worn etc.); and the perceiver (for example, face recognition ability etc.) may be important in determining the specific impact of face masks in different scenarios. We must consider in more detail *why* face masks impair performance. It may be that masks impair accuracy as they reduce the amount of local face information available to observers. Alternatively (or additionally), negative effects of face masks may arise from the disruption of normal holistic processing of faces (Tanaka & Farah, 1993). Thus, face mask research may also tell us more generally about face perception and recognition.

Further the effects of face masks on communication go beyond the direct effects associated with the attenuation of the acoustic signal, to the psychosocial and psychological realm. This especially true for people with hearing loss. It remains to be seen whether a transparent mask can be developed that is both visually and acoustically transparent which would remove the communication barriers that masks currently impose. We look forward to seeing the next wave of research considering the implications of face coverings for face perception and speech communication, topics that have both theoretical and applied interest.

## References

[CR1] Bennetts RJ, Johnson Humphrey P, Zielinska P (2022). Face masks versus sunglasses: Limited effects of time and individual differences in the ability to judge facial identity and social traits. Cognitive Research.

[CR2] Blazhenkova O, Dogerlioglu-Demir K, Booth RW (2022). Masked emotions: Do face mask patterns and colors affect the recognition of emotions?. Cognitive Research.

[CR3] Carlaw BN, Huebert AM, McNeely-White KL (2022). Detecting a familiar person behind the surgical mask: Recognition without identification among masked versus sunglasses-covered faces. Cognitive Research.

[CR4] Carragher DJ, Hancock PJB (2020). Surgical face masks impair human face matching performance for familiar and unfamiliar faces. Cognitive Research: Principles and Implications.

[CR5] Carragher DJ, Towler A, Mileva VR (2022). Masked face identification is improved by diagnostic feature training. Cognitive Research.

[CR6] Crinnion AM, Toscano JC, Toscano CM (2022). Effects of experience on recognition of speech produced with a face mask. Cognitive Research.

[CR7] Estudillo AJ, Wong HK (2022). Two face masks are better than one: Congruency effects in face matching. Cognitive Research.

[CR8] Freud E, Stajduhar A, Rosenbaum RS, Avidan G, Ganel T (2020). The COVID-19 pandemic masks the way people perceive faces. Scientific Reports-UK.

[CR9] Galvin KL, Tomlin D, Joubert L (2022). Effects of widespread community use of face masks on communication, participation, and quality of life in Australia during the COVID-19 pandemic. Cognitive Research.

[CR10] Ganel T, Goodale MA (2022). Smiling makes you look older, even when you wear a mask: The effect of face masks on age perception. Cognitive Research.

[CR11] Garcia-Marques T, Oliveira M, Nunes L (2022). That person is now with or without a mask: How encoding context modulates identity recognition. Cognitive Research.

[CR12] Grenville E, Dwyer DM (2022). Face masks have emotion-dependent dissociable effects on accuracy and confidence in identifying facial expressions of emotion. Cognitive Research.

[CR13] Gutierrez-Sigut E, Lamarche VM, Rowley K (2022). How do face masks impact communication amongst deaf/HoH people?. Cognitive Research.

[CR14] Gutz SE, Rowe HP, Tilton-Bolowsky VE (2022). Speaking with a KN95 face mask: A within-subjects study on speaker adaptation and strategies to improve intelligibility. Cognitive Research.

[CR15] Henke L, Guseva M, Wagemans K (2022). Surgical face masks do not impair the decoding of facial expressions of negative affect more severely in older than in younger adults. Cognitive Research.

[CR16] Hies O, Lewis MB (2022). Beyond the beauty of occlusion: Medical masks increase facial attractiveness more than other face coverings. Cognitive Research.

[CR17] Hsiao JH, Liao W, Tso RVY (2022). Impact of mask use on face recognition: an eye-tracking study. Cognitive Research.

[CR18] Kollenda D, de Haas B (2022). The influence of familiarity on memory for faces and mask wearing. Cognitive Research.

[CR19] Krishna A, Rodrigues J, Mitschke V (2021). Self-reported mask-related worrying reduces relative avoidance bias toward unmasked faces in individuals with low Covid19 anxiety syndrome. Cogn. Research.

[CR20] Lander K, Christie F, Bruce K (1999). The role of movement in the recognition of famous faces. Memory & Cognition.

[CR21] Lau WK, Chalupny J, Grote K (2022). How sign language expertise can influence the effects of face masks on non-linguistic characteristics. Cognitive Research.

[CR22] Lee E, Cormier K, Sharma A (2022). Face mask use in healthcare settings: Effects on communication, cognition, listening effort and strategies for amelioration. Cognitive Research.

[CR23] Manley KD, Chan JCK, Wells GL (2022). Improving face identification of mask-wearing individuals. Cognitive Research.

[CR24] McCrackin SD, Provencher S, Mendell E (2022). Transparent masks reduce the negative impact of opaque masks on understanding emotional states but not on sharing them. Cognitive Research.

[CR25] Pavlova MA, Sokolov AA (2022). Reading covered faces. Cerebral Cortex.

[CR26] Pazhoohi F, Kingstone A (2022). Unattractive faces are more attractive when the bottom-half is masked, an effect that reverses when the top-half is concealed. Cognitive Research.

[CR27] Poon BT, Jenstad LM (2022). Communication with face masks during the COVID-19 pandemic for adults with hearing loss. Cognitive Research.

[CR28] Rinck M, Primbs MA, Verpaalen IAM (2022). Face masks impair facial emotion recognition and induce specific emotion confusions. Cognitive Research.

[CR29] Sinagra C, Wiener S (2022). The perception of intonational and emotional speech prosody produced with and without a face mask: An exploratory individual differences study. Cognitive Research.

[CR30] Stajduhar A, Ganel T, Avidan G (2022). Face masks disrupt holistic processing and face perception in school-age children. Cognitive Research.

[CR31] Swain RH, O’Hare AJ, Brandley K (2022). Individual differences in social intelligence and perception of emotion expression of masked and unmasked faces. Cognitive Research.

[CR32] Tanaka JW, Farah MJ (1993). Parts and wholes in face recognition. Quarterly Journal of Experimental Psychology Section A.

[CR33] Thorley C, Acton B, Armstrong J (2022). Are estimates of faces’ ages less accurate when they wear sunglasses or face masks and do these disguises make it harder to later recognise the faces when undisguised?. Cognitive Research.

[CR34] Tso RV, Chui CO, Hsiao JH (2022). How does face mask in COVID-19 pandemic disrupt face learning and recognition in adults with autism spectrum disorder?. Cognitive Research.

[CR35] Wong HK, Estudillo AJ (2022). Face masks affect emotion categorisation, age estimation, recognition, and gender classification from faces. Cognitive Research.

