# Correction: Face coverings: considering the implications for face perception and speech communication

**DOI:** 10.1186/s41235-023-00490-1

**Published:** 2023-05-30

**Authors:** Karen Lander, Gabrielle H. Saunders

**Affiliations:** 1 Division of Psychology, Communication and Human Neuroscience, School of Health Sciences, University of Manchester, Manchester, M13 9PL UK; 2 Manchester Centre for Audiology and Deafness, and NIHR Manchester Biomedical Research Centre - Hearing Health Theme, University of Manchester, Manchester, UK

**Correction: Cognitive Research: Principles and Implications (2023) 8:24** 10.1186/s41235-023-00479-w

The original article [1] contained errors in co-author, Gabrielle H. Saunders’ name and affiliation which have both since been amended.
